# A computational simulation of the effect of hybrid treatment for thoracoabdominal aortic aneurysm on the hemodynamics of abdominal aorta

**DOI:** 10.1038/srep23801

**Published:** 2016-03-31

**Authors:** Jun Wen, Ding Yuan, Qingyuan Wang, Yao Hu, Jichun Zhao, Tinghui Zheng, Yubo Fan

**Affiliations:** 1 School of Civil Engineering and Architecture, Southwest University of Science and Technology, Mianyang, Sichuan, 621010, China; 2 Vascular Surgery Department of West China School of Medicine, Sichuan University, Chengdu, 610041, China; 3 Department of Applied Mechanics, Sichuan University, Chengdu, 610065, China; 4 Regenerative Medicine Research Center, West China Hospital, Sichuan University, Chengdu, 610041, China; 5 Key Laboratory for Biomechanics and Mechanobiology of Ministry of Education, School of Biological Science and Medical Engineering, Beihang University, Beijing, 100191, P. R. China

## Abstract

Hybrid visceral-renal debranching procedures with endovascular repair have been proposed as an appealing technique to treat conventional thoracoabdominal aortic aneurysm (TAAA). This approach, however, still remained controversial because of the non-physiological blood flow direction of its retrograde visceral revascularization (RVR) which is generally constructed from the aortic bifurcation or common iliac artery. The current study carried out the numerical simulation to investigate the effect of RVR on the hemodynamics of abdominal aorta. The results indicated that the inflow sites for the RVR have great impact on the hemodynamic performance. When RVR was from the distal aorta, the perfusion to visceral organs were adequate but the flow flux to the iliac artery significantly decreased and a complex disturbed flow field developed at the distal aorta, which endangered the aorta at high risk of aneurysm development. When RVR was from the right iliac artery, the abdominal aorta was not troubled with low WSS or disturbed flow, but the inadequate perfusion to the visceral organs reached up to 40% and low WSS and flow velocity predominated appeared at the right iliac artery and the grafts, which may result in the stenosis in grafts and aneurysm growth on the host iliac artery.

Thoracoabdominal aortic aneurysm (TAAA) is a continuous dilation of the descending thoracic aorta extending into the abdominal aorta. TAAAs are at high risk for rupture and death, the mortality is as high as 76% at 2 years if a large TAAA left unaddressed surgically^1^.

The treatment of TAAA is one of the most formidable challenges in cardiovascular surgery. Traditional open surgical repair of TAAA represents a complex surgical procedure with high morbidity and mortality, and it is still embarrassed in many high-risk patients with severe comorbidities despite the surgical developments^2^. In addition, although branched or fenestrated endovascular stents have been used to treat TAAA to preserve visceral perfusion, the experience is limited and these endovascular devices are still troublesome to manage and need to be refined^3^,^4^. Moreover, there is still controversy regarding the endovascular treatment for patients with TAAA of Marfan syndrome^5^.

Quiñones-Baldrich *et al*.^6^ first introduced a hybrid approach using both conventional and endovascular surgery to treat TAAA. This procedure has the following advantages: (1) avoids the open burden of thoractomy; (2) avoids the aortic cross-clamping to reduce visceral ischemia time, which influences the occurrence of severe complications such as renal impairment; (3) reduces the risk of neurological complications such as paraplegia and (or) paraparesis^7^. Hybrid repair of TAAA has been stepwise applied by several centers in the world since 1999. We searched the literature of TAAA hybrids from 1999 to 2014 using PubMed and this treatment was indicated the acceptable procedure for TAAA from the cumulate amounts (Fig. 1).

Because the site chosen for the origin of the visceral bypass grafts needs to allow for sufficient distal landing zone of the endovascular grafts, re-routing of abdominal aortic visceral branches are mostly constructed from the aortic bifurcation or common iliac artery^1^,^8^. Although satisfactory early and long term results for this hybrid TAAA repair has been achieved, the safety and durability of this retrograde visceral revascularization (RVR) have remained controversial, and some devastating complications such as visceral graft occlusion and pancreatitis are common in reports from literature^9^,^10^.

It has been suggested that specific hemodynamics or vessel wall constituents predispose this vessel portion to aneurysmal expansion and rupture^11^. Generally, an appropriate WSS value ranging from 4 to 20 dyne/cm^2^ is required to keep the normal cell morphology in the human blood vessels^12^. When WSS is low, the degeneration and apoptosis of endothelial cells happens, cell gaps gradually increase, the deposition of intraluminal thrombus (ILT) continues, and the adventitial integrity is gradually lost through inflammatory or ischemic changes^13^,^14^. Finally these pathologic changes promote the vessel wall dilation and degeneration and artery aneurysm develops, grows and rupture. It has also been suggested a connection between the spatial gradient of WSS and regions where aneurysm preferentially develops^15^. Furthermore, It is revealed that abdominal aortic aneurysm (AAA) rupture occurred at sites of predicted flow recirculation, where low WSS and thrombus deposition predominated^11^.

Retrograde blood flow through the bypass grafts likely induces the turbulence and disturbed flow that can raise the likelihood of graft failure or unfavorable patency^13^. In addition, based on the hemodynamic pathogenesis of TAAA, it is reasonable to speculate that the non-physiological blood flow direction resulting from the retrograde rerouting will possibly result in adverse effects on the distal abdominal aorta (AA) and likely induce some potential risks for patients’ long-term outcome^16^. However, to our knowledge, up to now there is no evidence to support the above hypothesis and no investigation on the hemodynamics of AA with RVR has been carried out.

In recent years, numerical investigations have been used increasingly by researchers seeking to understand vascular hemodynamics. Numerous researchers have shown that computational fluid dynamics can faithfully capture the physics of *in vitro* models. In addition, the computational techniques are able to provide a complete characterization of hemodynamic conditions including velocity profiles, WSS, and recirculation zones under precisely controlled conditions and obtain highly resolved data sets which are difficult to experimentally measure *in vitro* or *in vivo*^17^.

Therefore, this study aims to numerically make a comparison between a healthy AA and AA with RVR from the distal aorta or the common iliac artery in terms of hemodynamic parameters, so as to assess the ongoing performance of the AA and visceral grafts, evaluate whether the hybrid approach is amenable to amelioration and improvement, and provide a theoretical basis for the prevention of long-term complications.

## Results

Figure 2 presents the histograms of flow split to each branch artery of the AA before and after the RVR operation. As it revealed that the flow splits are totally different before and after the RVR operations. Moreover, the flow splits also differ significantly as the inflow site for RVR changes. Recall that, 70% of the blood that enters the healthy AA was prescribed to its visceral arteries and the remaining 30% flowed down the infrarenal segment through the bifurcation into the iliac arteries. However, 90.8% blood entering the supraceliac aorta was assigned to the digestive and renal arties, and the volume flowing down to the iliac arteries only accounted for 9.2% of the total inflow to the AA when RVR was constructed from the distal aorta. In addition, 100% blood entered the common iliac arteries for re-distribution which resulted in a more than two times increase of flow volume to the left iliac artery when RVR constructed from the right iliac artery. Specifically, for a healthy AA, RVR from the distal aorta, RVR from the right iliac artery, the percentages of blood flowing to the celiac artery were 21.5%, 19.4% and 14.21%, to the mesenteric artery were 16.1%, 23.8% and 17.58%, to the right renal artery were 16.1%, 23.8% and 9.85%, to the right renal artery were 16.1%, 23.8% and 10.37%, to the left iliac artery were 15%, 4.6% and 34.74%, to the right iliac artery were 15%, 4.6% and 13.21% respectively (Fig. 2).

Contour plots of WSS along AAs were displayed from anterior and posterior views in Fig. 3. For a healthy AA, areas of low WSSs (smaller than 2 dyne/cm^2^) were observed along the infrarenal section and the section opposite to the celiac and superior mesenteric arteries. In addition, its WSS distribution was quite non-uniform and high spatial oscillations in shear stress were present along the main body of AA, especially on the posterior wall of renal arteries, but shear stresses kept high on the iliac and visceral arteries (Fig. 3a,b).

The WSS distribution of AAs with RVR show distinct differences from that of the healthy AA, and different inflow sites for the visceral debranching resulted in different features. For both cases, WSSs were elevated and became uniform along the suprarenal section compared to those of a healthy AA. However, when RVR constructed from the distal aorta, the WSS became quite non-uniform on the host bed where the inflow site for the visceral bypasses located, namely, low and high WSS alternately appeared, and shear stresses significantly decreased on the wall of both iliac arteries while local high WSS region appeared from the anastomosis toe to the aortic bifurcation (Fig. 3c,d). When RVR established from the right iliac artery, it seems that the anastomosis region was not bothered with the low WSS problem, but the low and non-uniform WSS region shifted to the debranching graft and the downstream segment of the right iliac artery (Fig. 3e,f).

To have a closer look at the anastomotic region of RVR, Fig. 4 presents the zoom in view of the WSS distribution near the anastomosis. It was clear that the WSS level on the host aorta bed was not only low but also quite unevenly distributed when the grafts were sutured to the distal aorta. When RVR constructed from the right iliac artery, extreme low and oscillating WSS region appeared at the right iliac artery and its visceral grafts which were not true either for the healthy AA or the RVR from the distal aorta (Fig. 4).

To quantify the WSS level, the WSS distribution is plotted in Fig. 5 as a function of arc length along the posterior wall of the aorta. Examination of the WSS of a healthy AA revealed a violent fluctuation of its magnitude. First, the WSSs were no more than 2 dyne/cm^2^ at the distal aorta (arc length 0~4-cm), then gradually increased to approximately 4 dyne/cm^2^ and remained stable for a while (arc length 4~7-cm), the WSS experienced a sever oscillation in the neighborhood of the celiac, superior mesenteric and inferior mesenteric vessels. Three peak values and four troughs happened within arc length 7~12-cm. After the oscillation the WSS again rapidly decreased to zero value at superior mesenteric artery (arc length ~11-cm) and then gradually increased to 4 dyne/cm^2^ in the suprarenal aorta (arc length ~15-cm).

When RVR constructed from the distal aorta, the WSS oscillation happened within the section from the aortic bifurcation to the anastomosis toe (arc length 0~3 cm), and two peaks and three troughs happened. Generally speaking, the WSS value was higher than its corresponding value of the healthy AA, but the WSS approached zero at the anastomosis toe. Finally, the WSS gradually increased to a value of 5 dyne/cm^2^ approximately 6-cm distal to the bifurcation and kept stable up to the suprarenal aorta (arc length from 6~15-cm). When RVR constructed from the right iliac artery, WSS kept consistent high for the whole aorta section.

Figure 6 shows the streamlines superimposed the contour maps of velocity magnitude in the symmetry planes for three models, and the regions around the inflow site were zoomed in. a vortex developed at the renal artery of a healthy AA and the infrarenal aorta was a region with consistently low flow velocities relative to other sections. In addition, the velocity field in the infrarenal abdominal aorta was directed preferentially towards the anterior wall, and the flow to two iliac artery kept symmetry, laminar and parallel.

As shown in Fig. 6B,C the blood flow pattern at the inflow site of visceral debranching became quite disturbed and chaotic and vortices were observed and the orientation of the flow towards the anterior wall observed as a result of visceral graft outflows. Moreover, when fluid flowing from the host artery to the graft, the sudden geometry change shifted the fluid towards the outer wall of the graft under the action of circumferential forces and flow recirculation zones appeared near its inner wall. In addition, when RVR constructed from the right iliac artery, the blood flow near the inner wall of visceral grafts and toe were characterized by low-velocity recirculation flow and vortex pattern with flow direction opposite to the dominant flow way appeared (Fig. 6C).

## Discussion and Conclusion

The surgical treatment of the TAAA carries high mortality and morbidity rates despite various available surgical techniques. It has been suggested that the hybrid procedure has lower mortality and morbidity rates comparing to the open repair^18^,^19^. Majority patients in literatures underwent retrograde revascularization of visceral arteries from the aortic bifurcation or common iliac artery which is against physiological blood flow. This CFD study was the first to present the potential adverse effects of RVR in terms of hemodynamic performance.

According to the computational results, we can bring forth the following conclusion.

1. When RVR is constructed from the distal aorta, good news is that the flow split to visceral organs increased, bad news is that the flow flux to the iliac artery significantly reduced which only accounted for one third of the normal volume. Moreover, a complex and disturbed flow field developed at the distal aorta and flow recirculation, separation and stagnation, low WSS and sharp WSS gradients were observed.
2. When RVR is taken from the right iliac artery, good thing is that the whole segment of aorta was not troubled with low WSS or disturbed flow and the flow volume to the left iliac artery increased. However, benefits aside, the inadequate perfusion to the visceral organs raised and the ischemic volume reached up to 40%. In addition, although the flow split to the iliac right artery was only marginally decreased but the inflow site and parts of the graft segment were plagued with flow recirculation where low WSS and flow velocity predominated.
3. In a word, the inflow sites for the RVR have great impact on the hemodynamic performance of aortas with RVR. It is hard to say whether the distal aorta or the iliac artery is a better choice for the inflow site of bypass grafts, both sites have advantages and disadvantages. When RVR is from the distal aorta, the perfusion to visceral organs will be adequate but the infrarenal aorta is at high risk of aneurysm development. Moreover, we may expect the dilation of iliac artery and stenosis near the iliac bifurcation as a result of the known propensity of arteries to remodel outward in response to blood flow reductions. When RVR is from the right iliac artery, although the risk of infrarenal abdominal aneurysm reduced, the inadequate perfusion to visceral organs become unavoidable, the long-term patency of grafts may be shorten by stenosis and the host iliac artery is highly potential for aneurysm growth.

Based on the current research, we may have the following suggestion to the patients with hybrid TAAA surgery.

1. The location chosen for the proximal anastomosis.Being a man-made anastomosis between the aorta and graft, there is great chance to improve its performance and graft patency through optimal design^20^. As it shown that the location for the proximal anastomosis has great impact on the hemodynamic performance of AA after RVR operation, we raise concerns how to choose the optimal site of bypass inflow between infrarenal aorta and common iliac artery based on the patients actual situation, and it may help to avoid the potential risks for patients, including artery dilation or aneurysm, anastomotic stenosis, and visceral organ ischemia *et al*.
2. To avoid the stenosis of the distal anastomosis.Our results indicated that no matter the inflow site is on the aorta or the iliac artery, the anastomosis region is the most dangerous location for the initiation and the progression of AAA, it is fair to say that the quality of anastomosis decides the fate of the AA with RVR. The investigation of fluid dynamics in artery bypass graft (ABG) has pointed out that geometric factors including the anastomosis angle, the graft-to-host diameter ratio, surface irregularities and out-of-plane graft curvature are important parameters to affect the flow division in anastomoses and the hemodynamics in ABG^21^. In addition, it is reported that grafts featuring an extended patch or cuff may effectively provide a hood to ease the flow transition from the graft to the host which help to improve graft patency^22^. Furthermore, compliance mismatch has also been suggested as a cause for the bypass graft failure^23^. We believe that the hybrid TAAA surgery may refer to these research results and the postoperative performance of hybrid TAAA definitely will be improved by optimizing the anastomotic geometry and compliance match between graft and aorta.
3. Supervised lower limb exercise training is recommended to RVR patients.Previous *in vivo* studies using noninvasive medical imaging techniques to quantify blood flow indicated that lower limb exercise would multiply increase the total volumetric flows at the superaceliac aorta and iliac arteries^24^. As a result, the unfavorable hemodynamic conditions such as flow recirculation, low WSS, and high temporal oscillations in shear stress at the distal aorta of the RVV patients would be improved, and even light exercise of RVR patients would possibly protect himself against the development or progression of aneurysm^25^,^26^.
4. To guarantee regular follow-up.Patients should undergo the long regular follow-up for avoiding the related complications of stent graft or blood vessel prostheses or the potential dilation of infrarenal aorta or common iliac artery.

The current study provides information about the relationship between blood flow hemodynamics and inflow sites for visceral bypass that has not previously been reported, it help to evaluate the optimal inflow site for visceral rerouting to suit the individual needs and concerns for each patient and therefore help to guarantee long-term patency, safety and durability of RVR of hybrid TAAA surgery.

## Methods

### Model Geometry

**Figure 1:**
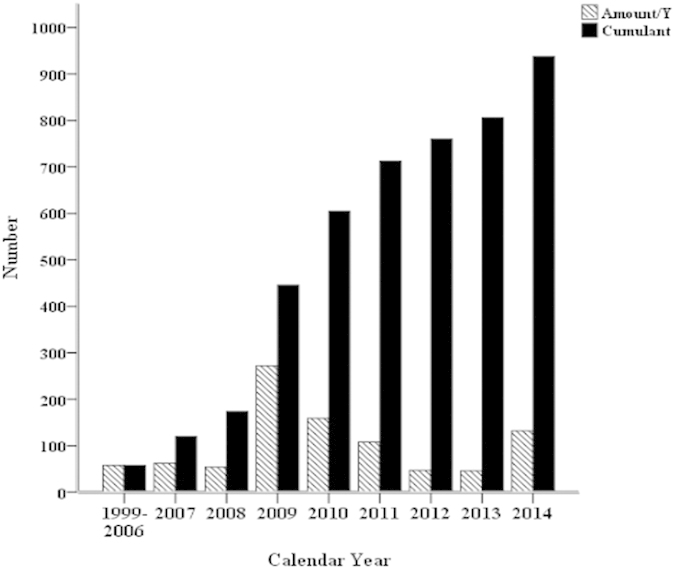
Estimated number of hybrid treatment for TAAA in the world from 1999 to 2014.

**Figure 2:**
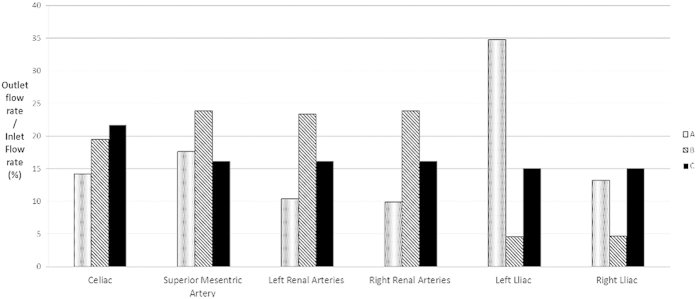
Histograms of the flow split for each branch artery before and after the RVR operation.

**Figure 3:**
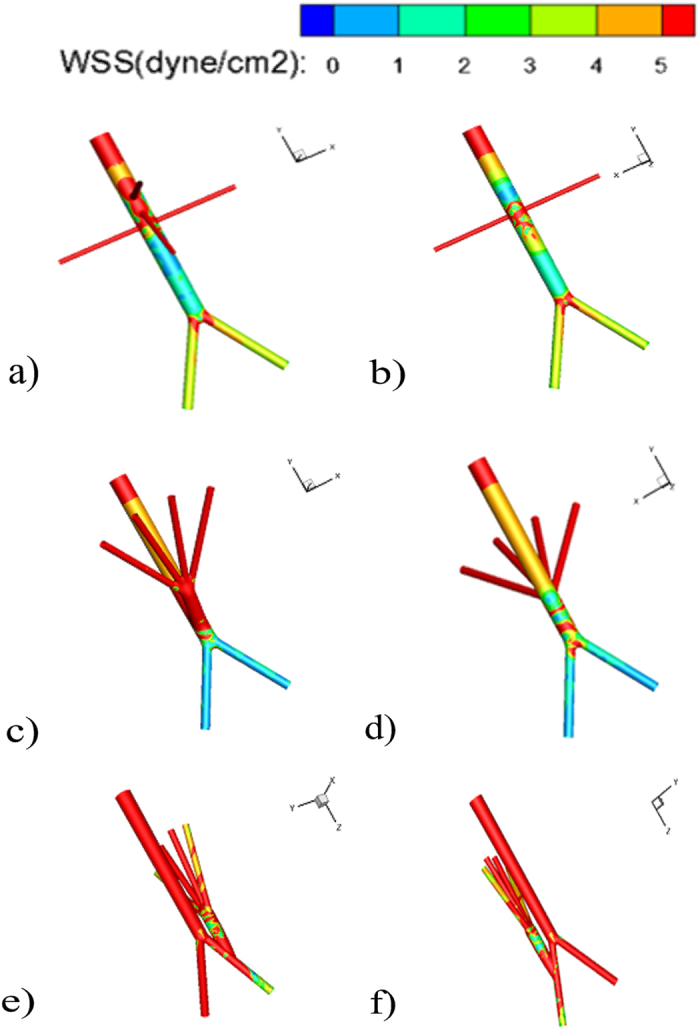
Contour plots of wall shear stress (WSS) along abdominal aorta are displayed from anterior and posterior views. (**a,b**) Healthy AA; (**c,d**) AA with RVR from the distal aorta; (**e,f**) AA with RVR from the right iliac artery.

**Figure 4:**
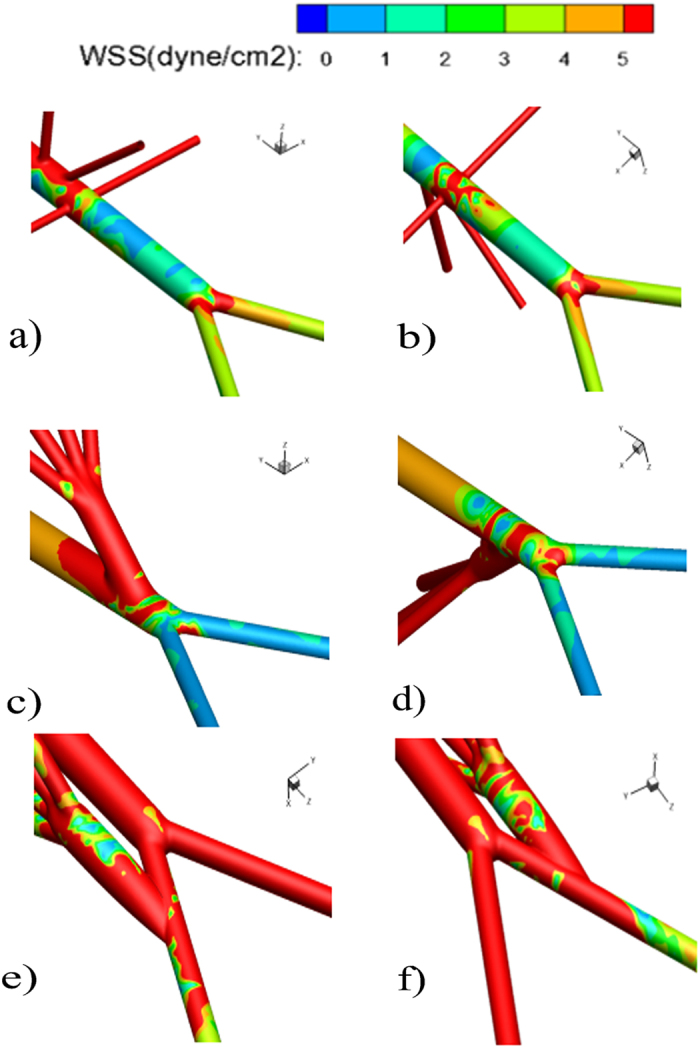
Zoom in wall shear stress (WSS) around the inflow site of grafts are displayed from anterior and posterior views. (**a,b**) Healthy AA; (**c,d**) AA with RVR from the distal aorta; (**e,f**) AA with RVR from the right iliac artery.

**Figure 5:**
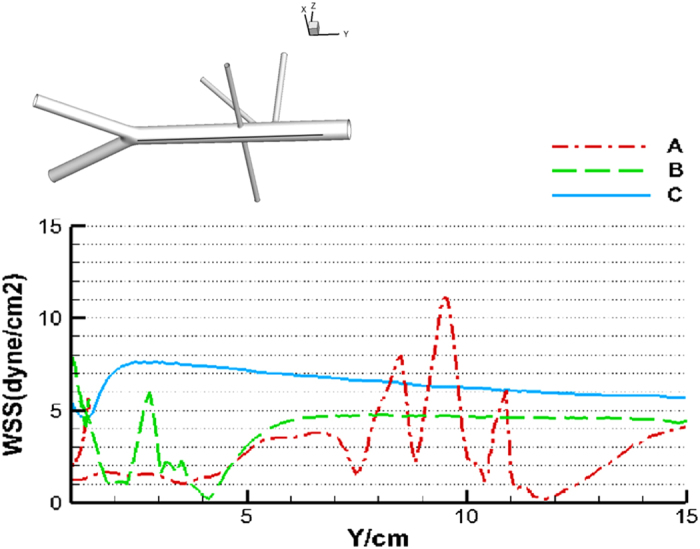
WSS as a function of arc length along the posterior wall from the diaphragm to the aortic bifurcation. (A) Healthy AA (arc length of ~15 cm); (B) AA with RVR from the distal aorta; (E,F) AA with RVR from the right iliac artery.

**Figure 6:**
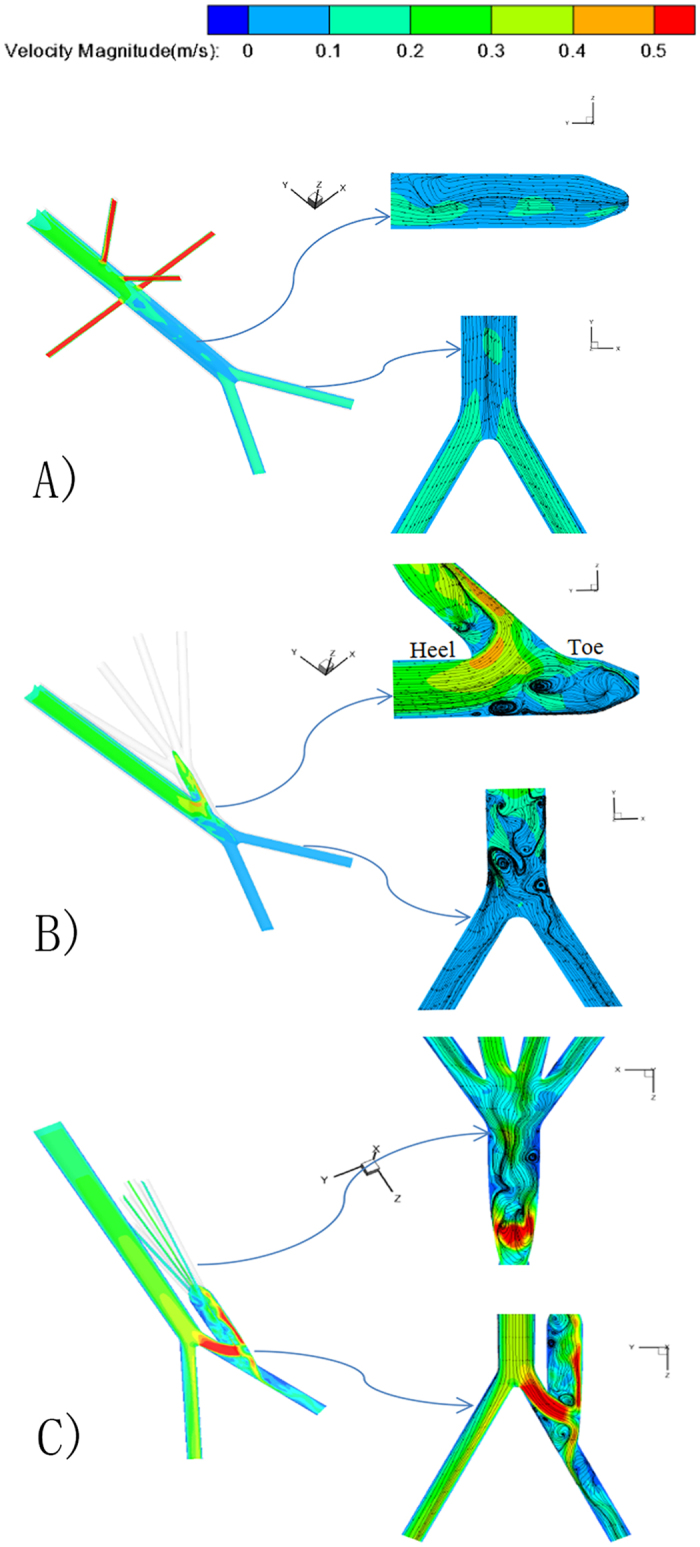
Midplane slice of the AA displaying contours of velocity magnitude superimposed with streamlines. Left: the overall model; right top: the longitudinal section to zoom in the anastomosis region; right bottom: the horizontal section to zoom in the iliac artery. (**A**) Healthy AA; (**B**) AA with RVR from the distal aorta; (**C**) AA with RVR from the right iliac artery.

**Figure 7:**
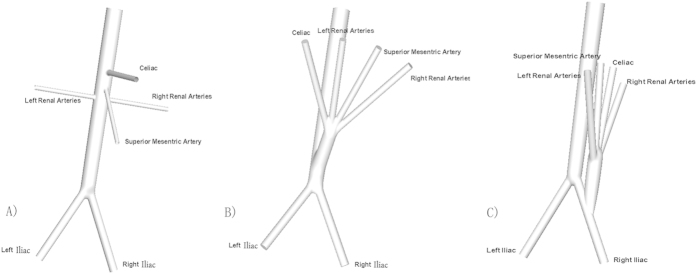
Sketches of model geometries. (**A**) Idealized healthy Abdominal aorta (AA) model with branches identified. Note the tapering of the aorta from the supraceliac aorta to the aortic bifurcation; (**B**) AA with retrograde visceral revascularization (RVR) from the distal aorta; (**C**) AA with RVR from the right iliac artery. Note that, to make a fair comparison, the geometry parameters of AA and iliac arteries for two models are kept consistent.

**Figure 8:**
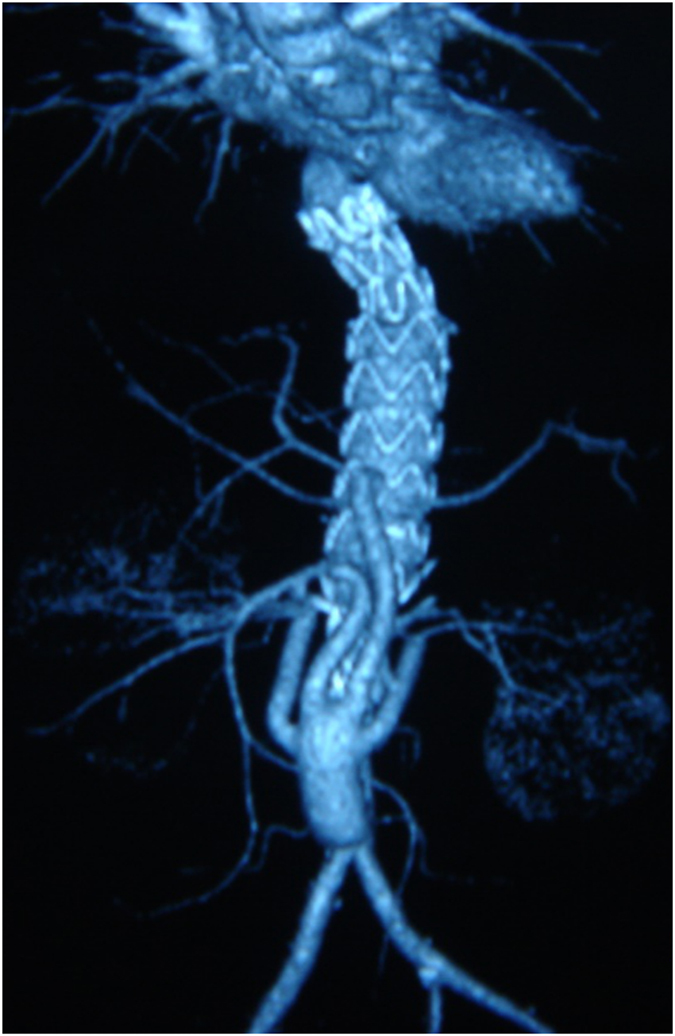
The postoperative CTA imaging of a patient with TAAA, which was underwent the hybrid treatment for TAAA. The quadr-furcated graft was reconstructed from the distal aorta to visceral arteries^27^.

The geometry was built using software PRO/E 5.0. First, the idealized healthy AA with visceral branches shown in Fig. 7A was constructed, the aorta tapers uniformly from a circular cross section with a diameter of 22-mm at the supraceliac aorta to a circular cross section with diameter of 16 mm at the aortic bifurcation. The take-off angles of both iliac arteries at the bifurcation are 30°. Diameters of common iliac arteries, superior mesentric artery, renal arteries, and celiac artery are 10-mm, 5-mm, 5-mm, and 6-mm respectively. Then based on the postoperative computer tomography angiography (CTA) of a patient with TAAA (Fig. 8), RVR from the distal aorta was constructed^27^. RVR from the right iliac artery was established referring to the literatures^2^,^28^. As shown in Fig. 1B,C the inverted quadr-furcated graft as donor vessels for debranching renal and visceral arteries was connected to the host artery, the diameters of the trunk and debranching grafts are 16-mm and 8-mm respectively. All other geometric parameters are consistent with those of the healthy AA.

### Governing Equations

As a preliminary study, the blood was assumed to be incompressible, laminar, homogenous and Newtonian, and the simulations were carried out under steady flow conditions. The corresponding governing equations are given as


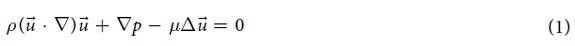



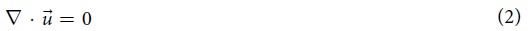


where 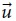 and *p* represent, respectively, the fluid velocity vector and the pressure. *ρ* and *μ* are the density of 1050 *kg*/*m*^3^ and dynamic viscosity of 3.5 × 10^−3^ *kg*/*m* · *s*.

### Boundary Conditions

The entrance boundary was defined as constant-velocity inlet of 0.18 *m/s*. According to Moore and Ku^29^, approximately 70% the blood flow from the supraceliac aorta split into the digestive and renal arties and the distributions are 31% to the celiac artery, 23% to the superior mesenteric artery, and 23% to each renal artery respectively, the remaining 30% flowing down the infrarenal segment was equally prescribed to the iliac arteries.

The flow rate in the AA models with RVR was mapped with the use of pressure boundaries. Due to the clinical fact that the perfusion pressure of visceral arteries are same if no artery stenosis happen, the perfusion pressure for the visceral organs kept unchanged before and after the operation, so assuming the pressure at the supraceliac artery was 100 mmHg, the outlet pressure at each visceral artery was then defined taking into account the corresponding pressure drop computed using the healthy AA model, the iliac outlet pressure was selected so that the entire flow rate through the model was equal to that of the healthy one.

The graft and vessel wall were assumed to be rigid and nonslip.

### Numerical Simulation

A finite volume mesh with 747,254 unstructured triangular elements and 208,613 nodes were generated using an automatic mesh generator ICEM (ANSYS Inc., Pittsburgh, PA). Meshes in the region of the aorta below the renal arteries, the aortic bifurcation and along the branch vessels were refined. In addition, a viscous layer of 0.1D was imposed adjacent to the vessel walls in order to capture the boundary layer.

The flow visualization and analysis were completed by the commercial CFD software Ansys FLUENT 12.0 which was based on the finite volume method. Default segregate implicit 3D solver was applied. Discretization of the equations involved a second order upwind differencing scheme, SIMPLE was adopted for the pressure velocity correction and the residual error convergence threshold was set as 1e-5.

## Additional Information

**How to cite this article**: Wen, J. *et al*. A computational simulation of the effect of hybrid treatment for thoracoabdominal aortic aneurysm on the hemodynamics of abdominal aorta. *Sci. Rep*. **6**, 23801; doi: 10.1038/srep23801 (2016).
